# Magnetic resonance imaging of uterine fibroids: a preliminary investigation into the usefulness of 3D-rendered images for surgical planning

**DOI:** 10.1186/s40064-015-1170-9

**Published:** 2015-07-28

**Authors:** Sayed Ahmad Zikri B Sayed Aluwee, Hiroki Kato, Xiangrong Zhou, Takeshi Hara, Hiroshi Fujita, Masayuki Kanematsu, Tatsuro Furui, Ryuichiro Yano, Nao Miyai, Ken-ichirou Morishige

**Affiliations:** Division of Regeneration and Advanced Medical Sciences, Department of Intelligent Image Information, Graduate School of Medicine, Gifu University, 1-1 Yanagido, Gifu, 501-1194 Japan; Department of Radiology, Gifu University School of Medicine, 1-1 Yanagido, Gifu, 501-1194 Japan; Department of Otolaryngology, Gifu University School of Medicine, Gifu, Japan

**Keywords:** Uterus, Fibroid, Magnetic resonance imaging, 3D

## Abstract

**Purpose:**

This study aimed to assess the efficacy of 3D surface-rendered (SR) magnetic resonance (MR) images for surgical planning of uterine fibroids.

**Methods:**

Ten patients with uterine fibroids underwent 3D volume isotropic turbo spin-echo acquisition (VISTA) sequences in sagittal planes. SR images showing the uterine body, endometrium, and fibroids were extracted from the raw MR data. The preoperative assessment for fertility-preserving fibroid enucleation was independently performed by two gynecologists using 2D sagittal and 3D SR images separately.

**Results:**

The required interpretation times [second] for sagittal versus SR images were 19.7 ± 9.5 versus 10.4 ± 5.1 for observer 1 (*p* < 0.05) and 47.5 ± 12.3 versus 19.7 ± 9.5 for observer 2 (*p* < 0.01). The accuracy rates of the planned surgical procedures from sagittal versus SR images were 50 versus 70% for observer 1 and 70 versus 70% for observer 2. The accuracy rates of the numbers of fibroids to be removed from sagittal versus SR images were 70 versus 80% for observer 1 and 70 versus 80% for observer 2.

**Conclusion:**

Compared with sagittal images, SR images could significantly reduce the time required for surgical planning of uterine fibroids without sacrificing the accuracy of the preoperative assessment.

## Background

The uterine fibroid represents the most common gynecologic and uterine neoplasm. Approximately 20–30% of women older than 35 years have uterine fibroids that are manifested clinically (Buttram and Reiter 1981). The most common clinical manifestations are pain and abnormal vaginal bleeding. Treatment options for patients with fibroids include medical regimens, surgery, and uterine artery embolization (UAE). The type of treatment depends on a variety of factors including the size, location and number of fibroids, medical history, and childbearing status. Myomectomy is a surgical procedure in which fibroids are removed while the uterus is preserved in women of child-bearing age (Myers et al. 2002). In several uncontrolled surgical trials, restoration of fertility after myomectomy has been reported, with pregnancy rates ranging between 44 and 62% (Berkeley et al. 1983; Gatti et al. 1989; Dubuisson and Chapron 1996; Dubuisson et al. 1996). The surgical procedures to remove uterine fibroids include abdominal myomectomy, laparoscopically assisted myomectomy (LAM), total laparoscopic myomectomy (TLM), transcervical resection (TCR).

Magnetic resonance (MR) imaging is the most accurate imaging technique for the detection and localization of uterine fibroids (Murase et al. 1999). It provides superior demonstration of uterine zonal anatomy and can be used to study an enlarged uterus that it is too large for ultrasonography. MR imaging also has an important role in the treatment of fibroids by assisting in surgical planning and monitoring the response to medical therapy. Although surgical simulation models using 3D surface or volume renderings based on computed tomography (CT) have been common in clinical practice for the upper abdominal organs, we were unable to find any reports of a surgical simulation model for uterine fibroids using 3D-rendered images based on MR imaging. Therefore, this study aimed to assess the efficacy of 3D-rendered images based on MR imaging for the surgical planning of uterine fibroids.

## Methods

### Patients

The study was approved by the human research committee of the institutional review board, and complied with the guidelines of the Health Insurance Portability and Accountability Act of 1996. The requirement for informed consent was waived because of the retrospective nature of this study. Between June 2013 and October 2013, 12 consecutive patients with suspected uterine fibroids underwent a 3D volume isotropic turbo spin-echo acquisition (VISTA) sequence using a 3.0-T unit. However, one patient with uterine adenomyosis and one with uterine fibroids after UAE were excluded from this study. In total, 10 patients with uterine fibroids (age range 39–54 years; average age, 47 years) were included in the study. Thirty-eight uterine fibroids (35 intramural, two subserosal, and one submucosal) were confirmed on MR images; the number of fibroids ranged from 1 to 10 (mean 3.8) per patient.

### MR imaging

All 10 patients were examined using a 3-T MR imaging system (Achieva Quasar Dual 3T; Philips Medical Systems, Best, The Netherlands). A phased-array body coil was used to obtain whole pelvic coverage. 3D VISTA (T2-weighted fast spin-echo imaging; TR/TE, 1,800/205 ms; imaging matrices, 512 × 512; field of view, 30 × 30 cm; section thickness/gap, 2/0 mm) images were obtained in sagittal planes. The number of slices and data acquisition time of the 3D VISTA imaging sequence including the uterine body and fibroids were 90 slices and 293 s, respectively.

### Anatomical structure segmentation and 3D surface rendering

**Fig. 1:**
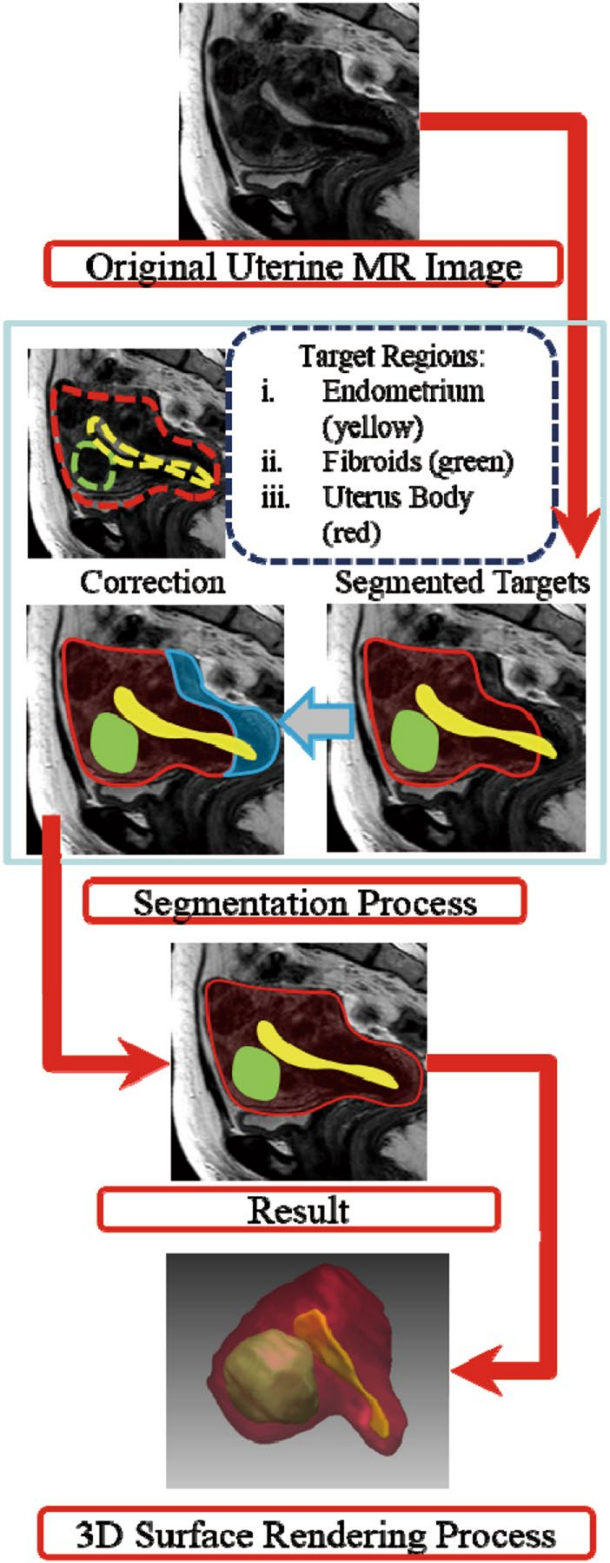
Process flow of the segmentation and 3D rendering. First, the target area (endometrium, fibroids, and uterine body) of the uterine MR images was segmented. Then, a correction process was applied to each slice from different image directions. Finally, a 3D surface rendering visualization was created.

The process flow for 3D surface-rendered (SR) images to demonstrate the shapes and spatial relations between the uterine body, endometrium, and multiple fibroids is shown in Fig. 1. As a pre-processing step before SR, the anatomical structures of the uterus need to be segmented from the MR images. This study used a semi-automatic approach to achieve the segmentation task. This approach requires the operator to trace the target region contours on a number of key 2D slices manually and then the computer estimates and reconstructs the whole 3D surface automatically. In the manual tracing step, the operator divided all the voxels on a 2D slice into two categories (target region and background) by drawing a closed curve on an MR slice. In the next step, a 2D interpolation function automatically creates the contour of the target organ on the adjacent slice by referring to and deforming the manually drawn contour. This interpolation process will continue to propagate until it reaches the next key slice that has a manual tracing contour from the operator. In the final step, validation and correction processes are applied to each of the 2D slices from different image directions. This process removes the false-positive parts of the pixels from the segmentation results. In this study, the manual tracing and final validation of the segmentation results were completed by an experienced radiologist (15 years of post-training experience in genitourinary imaging).

**Fig. 2:**
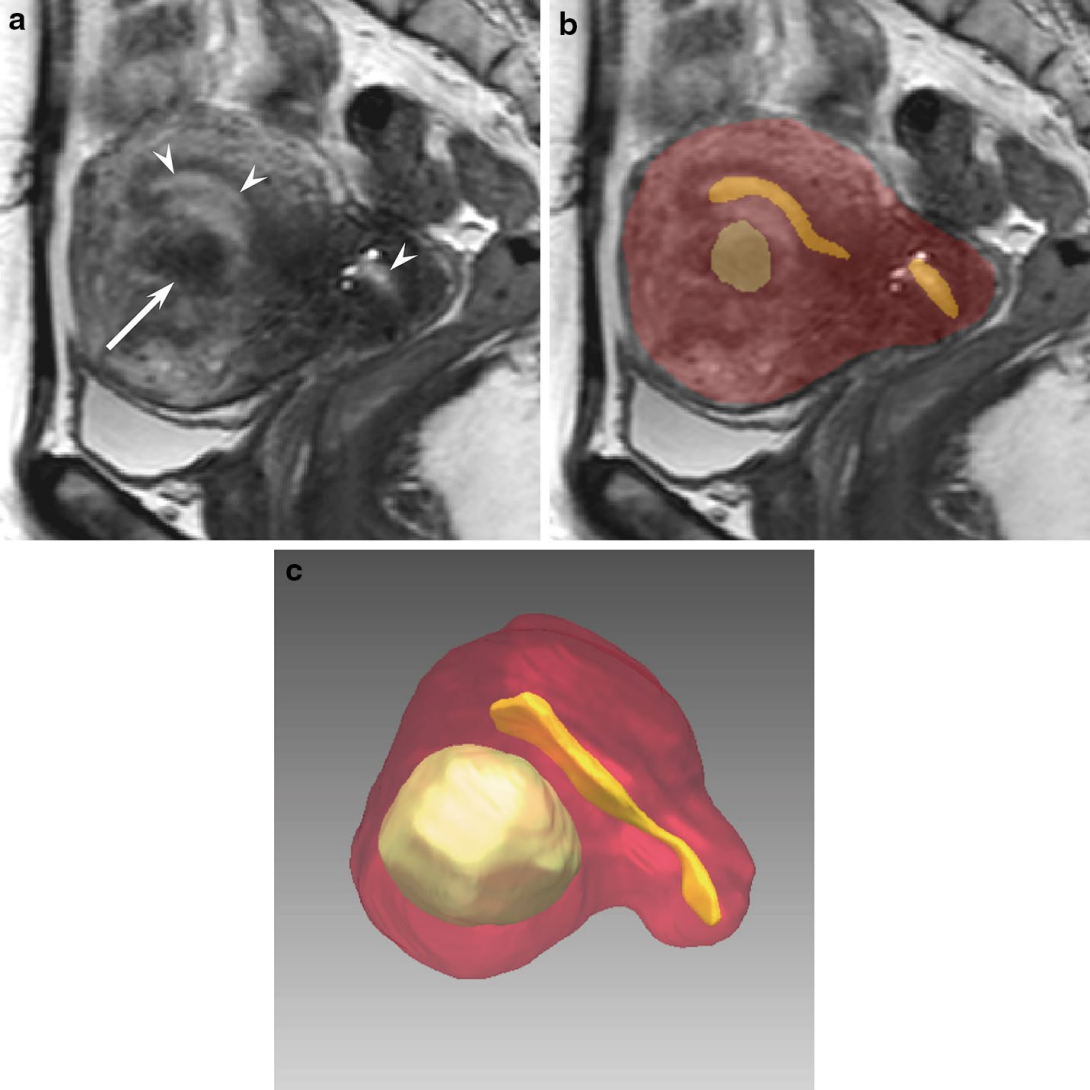
A 54-year-old woman with uterine fibroids (Case 6). The gold standard for the planned surgical procedure was total laparoscopic myomectomy (TLM). **a** A sagittal 3D VISTA image (TR/TE, 1,800/205 ms) demonstrates hyperintensity in the endometrium (*arrowheads*) and hypointensity in a fibroid (*arrow*). **b** The segmentation results of the target regions indicate the uterine body as a *red* color, the endometrium as a *yellow* color, and fibroids as a *green* color. **c** A 3D SR image precisely indicates the positional relationship of the uterine body (*red*), endometrium (*yellow*), and fibroids (*green*).

**Fig. 3:**
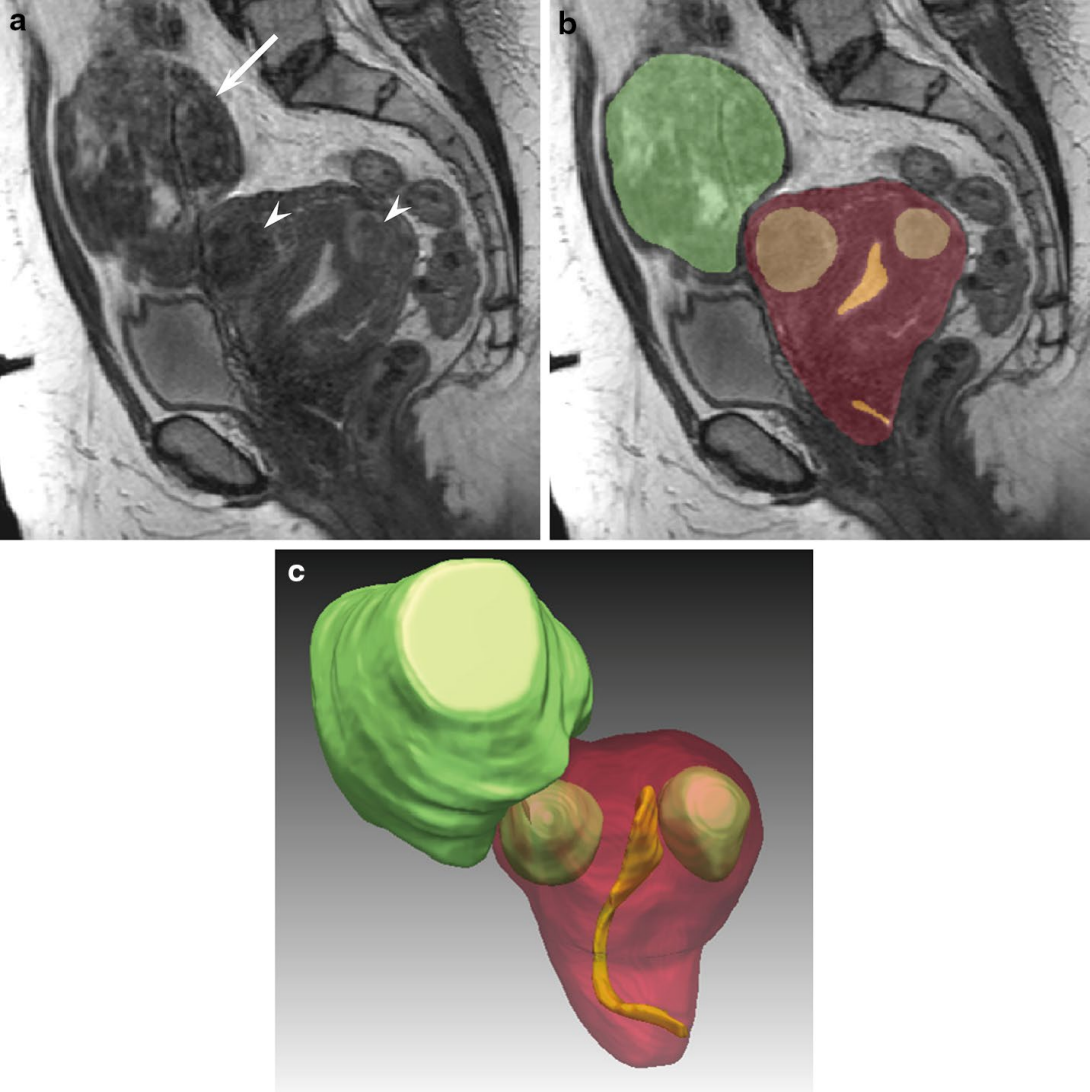
A 39-year-old woman with uterine fibroids (Case 1). The gold standard for the planned surgical procedure was laparoscopically assisted myomectomy (LAM). **a** A Sagittal 3D VISTA image (TR/TE, 1,800/205 ms) indicates a subserosal fibroid (*arrow*) and intramural fibroids (*arrowheads*). **b** The segmentation results of the target regions indicate the uterine body as a *red* color, the endometrium as a *yellow* color, and fibroids as a *green* color. **c** A 3D SR image precisely indicates the positional relationship of the uterine body (*red*), endometrium (*yellow*), and fibroids (*green*).

SR is an alternative to volume rendering used to create 3D reconstructions. SR is a means of extracting meaningful and intuitive information from 3D data sets and achieved by converting volume data into a surface representation (Shu et al. 1995). An extraction step and then conventional computer graphics techniques are used to render the surface. The surface extraction process is crucial; we used the marching cubes algorithm (Lorensen and Cline 1987) in addition to the smoothing method. The marching cubes algorithm can produce very high-quality images by generating a polygonal mesh of an isosurface from 3D voxels. For each segmented target, an explicit model is created to represent the target surface by using a number of polygons (Lorensen and Cline 1987). Examples of generated graphic models are shown in Figs. 2 and 3. The segmentation and rendering software application was implemented based on the medical imaging and interaction toolkit (MITK) open-source framework (Nolden et al. 2013).

### Image assessment

Two gynecologists (12 and 2 years of post-training experience in gynecological practice) unaware of patient age, symptoms, or medical history, independently reviewed all cases in a randomized fashion. The preoperative assessment for fertility-preserving fibroid enucleation was performed by referencing only sagittal images of the 10 patients. Subsequently, the same assessment was also performed referencing only the SR images. At first, the appropriate surgical procedures were chosen from among abdominal myomectomy, LAM, TLM, and TCR. Next, the number of fibroids to be removed was assessed. The required time for these surgical planning procedures was recorded. Finally, the degree of difficulty of surgical planning was scored using a four-point scale, where 1 represented very easy; 2, slightly easy; 3, slightly difficult; and 4, very difficult.

The gold standard for the planned surgical procedures and number of fibroids to be removed were defined by another experienced gynecologist (29 years of post-training experience in gynecological practice) referencing both sagittal and SR images. The accuracy rates of each observer were defined as the concordance rates with the gold standard.

### Statistical analyses

All statistical analyses were performed using SPSS version 18.0 (SPSS Inc., Chicago, IL, USA). The unpaired *t* test was used to compare the required time and difficulty scores between sagittal and SR images. Values of *p* < 0.05 were considered significant.

## Results

Our gold standard defined by an experienced gynecologist is summarized in Table 1. The planned surgeries included seven TLMs, two LAMs, and one TCR. Abdominal myomectomy was not chosen. The number of fibroids to be removed was 25 (22 intramural, two subserosal, and one submucosal) and ranged from 1 to 6 (mean 2.5) per patient. Thirteen intramural fibroids (size range 6–24 mm, mean size 15.2 mm), which were located within the myometrium of uterine body, were judged to be unnecessarily removed. The interpretations of two gynecologists are also summarized in Table 2.

**Table 1:** The gold standard for the planned surgical procedures and number of fibroids to be removed in ten cases

	Planned surgical procedure	Number of fibroids to be removed		
		Intramural	Subserosal	Submucosal
Case 1	LAM	3	1	0
Case 2	TLM	1	0	0
Case 3	LAM	1	0	0
Case 4	TLM	2	0	0
Case 5	TLM	1	0	0
Case 6	TLM	1	0	0
Case 7	TLM	5	1	0
Case 8	TLM	5	0	0
Case 9	TLM	3	0	0
Case 10	TCR	0	0	1

*LAM* laparoscopically assisted myomectomy, *TAM* total laparoscopic myomectomy, *TCR* transcervical resection.

**Table 2:** The results of gold standard and two gynecologists’ interpretations for the planned surgical procedures and number of fibroids to be removed in ten cases

	Planned surgical procedure			Number of fibroids to be removed		
	GS	O1 (Sagittal/SR)	O2 (Sagittal/SR)	GS	O1 (Sagittal/SR)	O2 (Sagittal/SR)
Case 1	LAM	TLM/TLM	AM/AM	4	4/4	3/4
Case 2	TLM	TLM/TLM	TCR/TLM	1	1/1	1/1
Case 3	LAM	TLM/TLM	AM/TLM	1	1/1	1/1
Case 4	TLM	TLM/TLM	TLM/TLM	2	2/2	2/2
Case 5	TLM	TLM/TLM	TLM/TCR	1	1/1	1/1
Case 6	TLM	TLM/TLM	TLM/TLM	1	1/1	1/1
Case 7	TLM	LAM/TLM	TLM/TLM	6	8/6	6/7
Case 8	TLM	LAM/LAM	TLM/TLM	5	9/6	7/6
Case 9	TLM	LAM/TLM	TLM/TLM	3	6/4	3/3
Case 10	TCR	TCR/TCR	TCR/TCR	1	1/1	2/1

*GS* gold standard, *O1* observer 1, *O2* observer 2, *Sagittal* sagittal images, *SR* surface rendered images, *AM* abdominal myomectomy, *LAM* laparoscopically assisted myomectomy, *TAM* total laparoscopic myomectomy, *TCR* transcervical resection.

The required times and difficulty scores for surgical planning are summarized in Table 3. The required times for sagittal versus SR images were 19.7 ± 9.5 s versus 10.4 ± 5.1 s for observer 1 (*p* < 0.05) and 47.5 ± 12.3 s versus 19.7 ± 9.5 s for observer 2 (*p* < 0.01). The difficulty scores for sagittal versus SR images were 1.6 ± 0.7 versus 1.4 ± 0.7 for observer 1 (*p* = 0.53) and 2.7 ± 0.7 versus 2.4 ± 0.5 for observer 2 (*p* = 0.28).

**Table 3:** The required time and difficulty score for the surgical planning by use of two methods in each observer

	Observer 1			Observer 2		
	Sagittal	SR	*p* value	Sagittal	SR	*p* value
Required time^a^	19.7 ± 9.5	10.4 ± 5.1	<0.05*	47.5 ± 12.3	19.7 ± 9.5	<0.01*
Difficulty score	1.6 ± 0.7	1.4 ± 0.7	0.53	2.7 ± 0.7	2.4 ± 0.5	0.28

*Sagittal* sagittal images, *SR* surface rendered images.

* The required time of SR is significantly shorter than that of sagittal.

^a^The unit of required time is second.

The accuracy rates of the planned surgical procedures and numbers of fibroids to be removed are summarized in Table 4. The accuracy rates of the planned surgical procedures from sagittal versus SR images were 50 versus 70% for observer 1 and 70 versus 70% for observer 2. The accuracy rates of the number of fibroids to be removed from sagittal versus SR images were 70 versus 80% for observer 1 and 70 versus 80% for observer 2.

**Table 4:** The accuracy rates of planned surgical procedures and number of fibroids to be removed for two methods in each observer

	Observer 1		Observer 2	
	Sagittal	SR	Sagittal	SR
Surgical procedures	50 (5/10)	70 (7/10)	70 (7/10)	70 (7/10)
Number of fibroids	70 (7/10)	80 (8/10)	70 (7/10)	80 (8/10)

In surgical procedures and number of fibroids, data are percentages and numbers in parentheses are numbers of patients.

*Sagittal* sagittal images, *SR* surface rendered images.

## Discussion

Uterine fibroids are the most common uterine neoplasms and involve smooth muscles with varying amounts of fibrous connective tissue. As fibroids enlarge, they may outgrow their blood supply, leading to various types of degeneration such as hyaline, myxoid, cystic, red degeneration and dystrophic calcification. Although fibroids typically appear as well-circumscribed masses of decreased signal intensity on T2-weighted images, they can have a variable appearance depending on the presence of cystic degeneration, necrosis, hemorrhage, and cellular-type leiomyoma (Murase et al. 1999; Szklaruk et al. 2003; Fasih et al. 2008; Ueda et al. 1999). The roles of MR imaging for patients with fibroids include differentiation from other pathologies, tumor localization, prediction of treatment outcome, and monitoring after therapy (Imaoka et al. 2003).

Compared with 2D T2-weighted imaging, advantages offered by 3D T2-weighted imaging include a higher signal-to-noise ratio (SNR), greater tumor conspicuity, fewer artifacts, and the ability to perform multi-planar reformation; thus, it has the potential to improve the performance of MR imaging in the evaluation of uterine carcinoma (Hori et al. 2011). Although the tumor conspicuity of fibroids was significantly worse on 3D T2-weighted images than on 2D T2-weighted images at 3.0 T in a previous study (Hori et al. 2011), it may be possible that better image contrast can be achieved by adjusting imaging parameters.

MR imaging is commonly used to help determine the treatment plan for uterine fibroids, especially when the surgical intervention being considered is myomectomy. However, it is not easy for trainees and medical students to understand the complicated signal intensities and characteristics of MR images. Because sagittal images are shown as 2D models, practical experience is required to develop spatial recognition skills from sagittal images. On the other hand, because SR images are shown as 3D models, spatial recognition becomes stunningly easy, especially for trainees and medical students. The 3D visualization of a high-quality SR image requires accurate segmentation results. Understanding and segmenting uterine structures on 2D MR images is a challenging task because of the large variety of shapes, positions, symptoms, patient histories and poor image resolutions of MR images. Our method can provide satisfactory segmentation results and generate natural 3D views that show uterine structures from different directions, permit scaling, and allow operators to adjust the transparency to view structures inside the uterus clearly. In our series, SR models significantly reduced the time required for surgical planning of uterine fibroids in comparison with sagittal images, and this phenomenon was especially prominent in the less-experienced gynecologist.

**Fig. 4:**
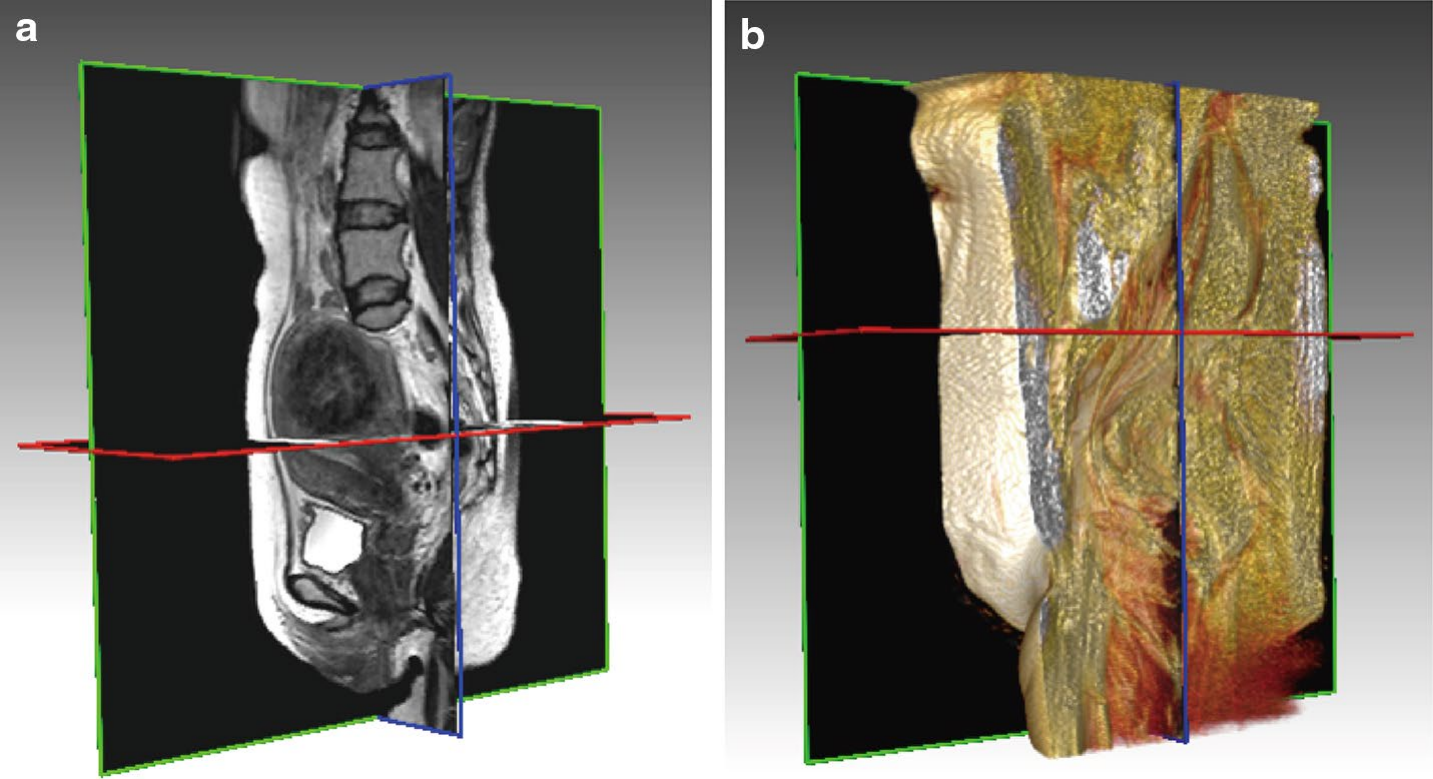
Advanced visualization by combining 2D slice and 3D rendering views. **a** 2D cross-sectional views of uterine MR images provide the advantage of viewing 2D images in different planes. **b** A combination of 2D and 3D views to provide visualization of the uterine structures from different directions and with scaling. The positional relationship of the uterine structures with respect to others organ nearby and the types of fibroids can be understood easily with the combination.

The present study has several limitations. First, because the study was conducted at a single institution, the cohort was small. Second, although subjects were administered intramuscular butyl scopolamine to prevent peristalsis artifacts, peristalsis artifacts were observed in some cases. Third, because we used semi-automatic segmentation methods that need to be corrected manually slice by slice, it took at least 1 h to correct the segmentation of each patient’s data before making SR images. Therefore, we need to improve the semi-automatic segmentation method and develop a fully-automatic segmentation method to reduce the processing time for generating graphic models. In the future, we plan to incorporate 3D volume rendering visualization and 2D section views with MR images as shown in Fig. 4a, b, respectively. It is sure that the total time for preparing and using 3D views was longer than viewing 2D raw data now, however our approach (engineers, radiologists, and surgeons worked together) potentially leads to a better results and gives the convenience to surgeons who are always busy on the operations. We are improving our system by providing more useful information instead of only showing the 3D surfaces.

## Conclusion

In conclusion, 3D-rendered images could significantly reduce the time required for surgical planning of uterine fibroids without sacrificing the accuracy of the preoperative assessment in comparison with sagittal images. By using 3D-rendered images, spatial recognition becomes stunningly easy, especially for less-experienced gynecologists. Therefore, 3D-rendered images might be useful for education and may aid in reducing the burden on gynecologists.

## Abbreviations

MR: magnetic resonance
SR: surface-rendered
VISTA: volume isotropic turbo spin-echo acquisition
UAE: uterine artery embolization
LAM: laparoscopically assisted myomectomy
TLM: total laparoscopic myomectomy
TCR: transcervical resection
CT: computed tomography
MITK: medical imaging and interaction toolkit
SNR: signal-to-noise ratio
